# The health and well-being of children with medical complexity and their parents’ when admitted to inpatient care units: A scoping review

**DOI:** 10.1177/13674935241312299

**Published:** 2025-01-29

**Authors:** Lyndsay Mackay, Tammie Dewan, Lauren Asaad, Francine Buchanan, K Alix Hayden, Lara Montgomery, Una Chang

**Affiliations:** 1 School of Nursing, 4402 Trinity Western University, Langley, BC, Canada; 2 Department of Pediatrics, 2129 University of Calgary, Calgary, AB, Canada; 3 9978 Alberta Children’s Hospital, Calgary, AB, Canada; 4 7979 The Hospital for Sick Children, Toronto, ON, Canada; 5 Library and Cultural Resources, 2129 University of Calgary, Calgary, AB, Canada

**Keywords:** Child, hospitalized, child, medically fragile, children with disabilities, scoping review, well-being

## Abstract

Children with medical complexity (CMC) are regularly admitted to inpatient care units to receive medical care. While admissions for CMC and their parents can negatively impact their health and well-being, mapping of evidence in this area appears underreported. A scoping review using the Joanna Briggs Institute methodology was conducted to map evidence on CMC and parents’ experiences of care. The purpose of this paper is to report the findings from the scoping review specific to CMC and parents’ experiences of care on their health and well-being. A total of 24 articles were synthesized, and themes included: psychological impacts for parents, impacts on functions of daily living, parents’ coping strategies for psychological well-being, impacts of hospitalization on CMC, CMC coping strategies, spirituality, and interventional studies. Findings from this review demonstrate that CMC and their parents struggled with their psychological and emotional well-being and that both CMC and parents coped with the stress of hospitalization in a variety of ways. Healthcare professionals need to be educated on how to support CMC and their parents during hospitalization. Future development and implementation of innovative care models and interventions that offer CMC and their parents with enhanced psychosocial support are recommended.

## Introduction

Children with medical complexity (CMC) are regularly admitted to inpatient care units because they require multiple procedures, surgeries, and management by highly skilled and specialized healthcare professionals (HCP) (Berry et al., 2013). Hospital admissions can be a significant time of stress for both CMC and their parents because care provided to CMC is fragmented and lacks comprehensive care coordination (Dewan et al., 2022).

CMC have intensive care needs and medical fragility, often distinguished by congenital and/or acquired multisystem diseases, neurologic conditions, functional impairments, and a requirement of medical technology for survival and activities of daily living (Cohen et al., 2011, 2018). CMC consist of four key characteristics: multiple complex chronic diseases, high healthcare utilization, functional limitations, and high caregiving needs (Cohen et al., 2011, 2018; Dewan and Cohen, 2013).

The population of CMC is significantly growing in size due to advances in medical and surgical care that have increased their survival (Orkin et al., 2020), which is accompanied by increased hospitalization rates for CMC (Burns et al., 2010). Due to their complex medical needs and frequent hospitalizations, CMC accrue one-third of pediatric costs to healthcare systems (Cohen et al., 2012) and 41% of pediatric hospital expenditures (Bergman et al., 2020; Simon et al., 2010; Srivastava et al., 2016).

Parents are responsible for providing and managing care to CMC, including providing basic and medical care (Mandic et al., 2017). Caregiving for CMC is unpredictable and uncontrollable in nature (Murphy et al., 2007), and parents do not have alternatives to the all-consuming care they must provide to their child (Kirk, 2001). Consequently, parents of CMC experience emotional burden and increased risk for clinical depression from having to provide life-sustaining care to their child (Carnevale et al., 2006; Cockett, 2012; Toly et al., 2012).

Care provided to CMC on inpatient care units is often disjointed and lacks continuity (Leary et al., 2020; Lin et al., 2020). CMC experience frequent medical errors (Blaine et al., 2022; Stockwell et al., 2018; Weingart et al., 2000), poor health outcomes (Whiting, 2014; Woodgate et al., 2015), and re-admissions (Cohen et al., 2012) when admitted to inpatient care units. Parents of CMC experience difficulties negotiating their role in the hospital as current care models do not always incorporate parent expertise or distinctly delineate the roles of team members, including family members (Dewan et al., 2022). Thus, CMC and their parents are at risk for altered health and well-being due to children’s complex care demands and navigating healthcare systems. It is advantageous to develop new and innovative models of care specific to CMC inpatient care. To do this, an in-depth understanding of CMC and their parents’ experiences of care is required. A clear understanding of CMC and their parents’ experiences of care will equip HCP, researchers, and healthcare systems to tailor and personalize bedside care, inform research, and guide policy development (Oben, 2020).

### Aim

To report synthesized findings on CMC and parents’ experiences while receiving inpatient care that are related to their general health and well-being.

## Methods

### Approach

This article is the second of two papers reporting results from a scoping review conducted in accordance with the Joanna Briggs Institute (JBI) methodology for scoping reviews and reported in accordance with Preferred Reporting Items for Systematic Reviews and Meta-Analysis extension for Scoping Reviews checklist (see supplemental file 1) (Peters et al., 2015, 2020; Tricco et al., 2018). The scoping review was conducted to identify, map, and synthesize available evidence on experiences of inpatient care for CMC and their parents. Given the large number and complexity of included studies, it was decided to report findings in two separate articles. The aim of the first published article was to report findings related to the CMC and their parents’ experiences of healthcare services as a “user” or “consumer” (Dewan et al., 2024). The aim of this review article is to report findings specific to CMC and their parents’ experiences of receiving inpatient care related to their health and well-being. The scoping review protocol was registered with Open Science Framework (registration number: 10.17605/OSF.IO/CP4MX).

### Search strategy

An academic health sciences librarian (KAH) constructed the search in the following databases: EMBASE (OVID), Cumulative Index of Nursing and Allied Health Literature Plus with Full Text (EBSCO), Web of Science Core Collection, MEDLINE(R) and Epub Ahead of Print, In-Process, In-Data-Review & Other Non-Indexed Citations and Daily (OVID), and APA PsycInfo (OVID). CMC and inpatient care setting were the two main concepts from which keywords and subject headings were developed. Searches were conducted on April 26, 2022 and limited to January 1, 2000 and onwards. A 20 year time limit was applied because medical care for CMC has changed significantly in the past 20+ years. See supplemental file 2 for complete search strategies.

### Inclusion criteria

#### Participants

This review included CMC and their parents. CMC are defined as a heterogenous group with the following: (a) multiple chronic conditions, (b) high utilization of healthcare services, (c) high care needs, and (d) functional limitations. CMC were between the ages of 1–18, and a minimum of 50% of participants in each research article had to be considered CMC or a parent of a CMC. Parents included biological, adoptive, and foster parents or legal guardians.

#### Concept

The concept of focus for this review was CMC and parents’ humanistic and illness-related experiences of care when admitted to inpatient care units, which comprises physical, mental, and social dimensions of health and well-being (Oben, 2020). Experience of care is defined as the collective interactions shaped by hospital organizational culture and is influenced by patient and family perceptions (Oben, 2020).

#### Context

This scoping review included articles specific to inpatient units at hospitals, including both inpatient wards and pediatric intensive care units (PICU). Excluded settings included: neonatal intensive care units, outpatient/ambulatory clinics, rehabilitation services, and palliative care.

#### Types of sources

Peer-reviewed quantitative, qualitative, mixed-methods, and reviews were included. Grey literature was not searched or included.

### Evidence selection

Following the search, all identified records were collated and uploaded into Covidence (Veritas Health Innovation, 2020), and duplicates were removed. Four reviewers (LJM, DW, LA, and FB) piloted tested inclusion criteria on 50 random titles and abstracts until a minimum of 90% inter-rater reliability was attained. All titles and abstracts were then reviewed against inclusion criteria by at least two independent reviewers (LJM, TD, LA, and FB). All potential articles were retrieved in full text and assessed by two independent reviewers for possible inclusion (LJM, TD, and LA). Reasons for exclusion were documented. Disagreements at any stage were resolved through discussion.

### Data extraction

Data were extracted by one reviewer from selected papers into a modified draft version of the JBI data extraction instrument (Peters et al., 2020) and verified by a second reviewer (LJM, LA, and LM). Extracted data included publication details, study design and details (i.e., purpose, setting, sample size, participant characteristics, concepts, and measurement tools/outcome measures), and main findings. Study findings specific to CMC and parents’ human and illness experiences were grouped together, and findings specific to CMC and their parents’ experiences receiving healthcare as consumers were placed in another group. Quality appraisals are not mandatory within the JBI methodology for scoping review; however, they are useful to identify and report risk of bias (Peters et al., 2015, 2020; Tricco et al., 2018). Quality appraisals were not completed for articles in this scoping review.

### Data presentation and analysis

Evidence was descriptively mapped and presented in tables and figures using frequency counts of the following: author, date, country, study purpose, sample characteristics, study method, key concepts, measurement tools/outcome measures, and main study results. Since the majority of included studies specific to health and well-being were qualitative in nature, thematic analysis was conducted by two reviewers (LJM and LM) (Braun and Clarke, 2006). Data from included studies were initially coded, and codes were compared and contrasted, looking for similarities and differences. Groups of coded data were developed and labeled as themes.

## Findings

### Overview of studies

**Figure 1.**
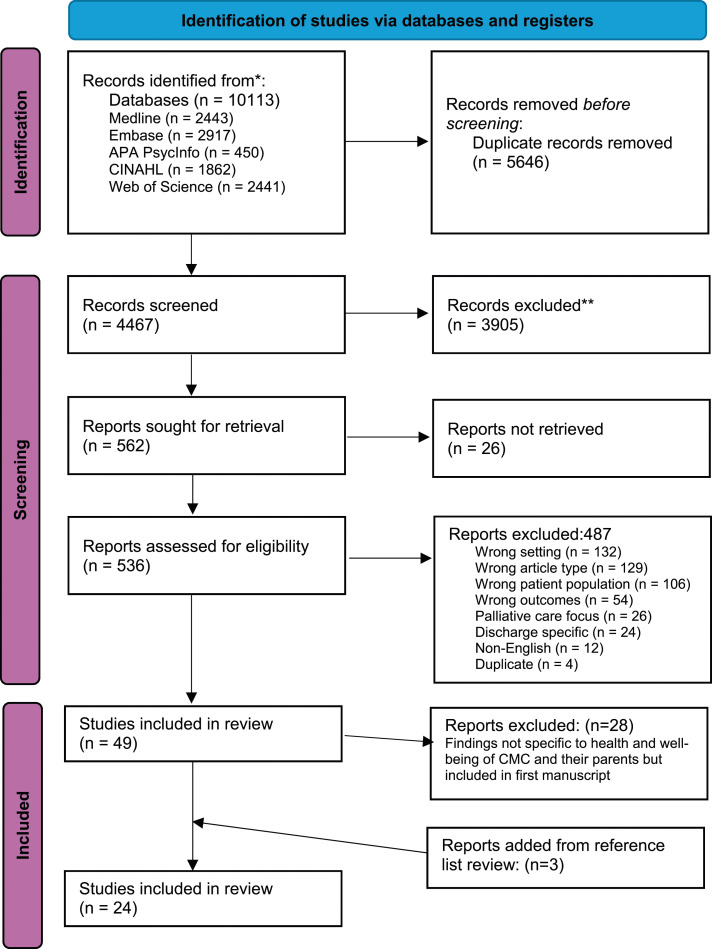
PRISMA flow diagram.

A total of 4467 titles and abstracts were screened against inclusion criteria. Figure 1 presents a Preferred Reporting Items for Systematic Reviews and Meta-Analysis flow diagram outlining the search and screening process. A total of 49 studies were included in the large scoping review. Findings from 21 of those articles were included in this synthesis because they had findings specific to health and well-being of CMC and their parents. After reviewing reference lists of included reviews, three articles were added, resulting in a total of 24 articles included in this review. Fourteen studies were qualitative, three studies were quantitative, five mixed-methods, and two studies were review articles. See Table 1 for numerical summary of included studies and Table 2 for overview of included studies.

**Table 1.** Numerical summary of studies.

Characteristics	No. of studies	Studies
Country		
Australia	2	Mimmo et al. (2019); Phua et al. (2005)
Canada	1	So et al. (2014)
Germany	1	Engler et al. (2020)
Jordan	1	Atout et al. (2017)
Malaysia	1	Taib et al. (2021)
Sweden	1	Hagvall et al. (2016)
Switzerland	2	Grandjean et al. (2021); Seliner et al. (2016)
United Kingdom	6	Oulton et al. (2018, 2020); Bull and Gillies (2007); Noyes (2000); Sexton et al. (2005); Shilling et al. (2012)
United States	11	Bay and Algase (1999); Borschuk et al. (2021); Brady et al. (2020); Goodnite (2013); Henderson et al. (2017); Madrigal et al. (2016); Nolan et al. (2014); Ridner (2004); Woodson et al. (2015); Wright-Sexton et al. (2020)
Participants		
Mothers	1	Atout et al. (2017)
Parents/Parents	17	Borschuk et al. (2021); Brady et al. (2020); Engler et al. (2020); Grandjean et al. (2021); Hagvall et al. (2016); Henderson et al. (2017); Madrigal et al. (2016); Nolan et al. (2014); Noyes (2000); Oulton et al. (2020); Phua et al. (2005); Seliner et al. (2016); Sexton et al. (2005); So et al. (2014); Taib et al. (2021); Woodson et al. (2015); Wright-Sexton et al. (2020)
Health care professionals	4	Atout et al. (2017); Henderson et al. (2017); Nolan et al. (2014); Wright-Sexton et al. (2020)
Children	3	Bull and Gillies (2007); Noyes (2000); Oulton et al. (2018)
Undergraduate students	1	Borkovec et al. (1983)
Not specified (review/Concept analysis)	5	Bay and Algase (1999); Goodnite (2013); Mimmo et al. (2019); Ridner (2004); Shilling et al. (2012)
Diagnosis		
Not specified	7	Bay and Algase (1999); Bull and Gillies (2007); Goodnite (2013); Hagvall et al. (2016); Henderson et al. (2017); Madrigal et al. (2016); Ridner (2003)
Neurological/Neurodevelopmental	14	Atout et al. (2017); Brady et al. (2020); Engler et al. (2020); Grandjean et al. (2021); Mimmo et al. (2019); Nolan et al. (2014); Oulton et al. (2018, 2020); Phua et al. (2005); Seliner et al. (2016); Shilling et al. (2012); Taib et al. (2021); Woodson et al. (2015); Wright-Sexton et al. (2020)
Nephrology	3	Atout et al. (2017); Shilling et al. (2012); So et al. (2014)
Hepatic	1	So et al. (2014)
Cardiovascular	6	Atout et al. (2017); Engler et al. (2020); Grandjean et al. (2021); Nolan et al. (2014); Woodson et al. (2015); Wright-Sexton et al. (2020)
Respiratory	4	Engler et al. (2020); Grandjean et al. (2021); Nolan et al. (2014); Wright-Sexton et al. (2020)
Gastrointestinal	5	Grandjean et al. (2021); Nolan et al. (2014); Shilling et al. (2012); So et al. (2014); Woodson et al. (2015)
Metabolic	2	Engler et al. (2020); Seliner et al. (2016)
Oncology	2	Grandjean et al. (2021); Woodson et al. (2015)
Mental health	1	Borkovec et al. (1983)
Ventilator-dependent	2	Borschuk et al. (2021); Noyes (2000)
Genetic/Chromosomal	4	Engler et al. (2020); Nolan et al. (2014); Seliner et al. (2016); Wright-Sexton et al. (2020)
Parenteral nutrition	1	Sexton et al. (2005)
Learning disability	1	Shilling et al. (2012)
Transplant	1	Woodson et al. (2015)
Hematological	1	Woodson et al. (2015)
Orthopedic	1	Woodson et al. (2015)
Study design		
Qualitative	15	
*Case Study*	1	Atout et al. (2017)
*Concept Analysis*	2	Bay and Algase (1999); Ridner (2004)
*Thematic Analysis*	2	Brady et al. (2020); Sexton et al. (2005)
*Grounded Theory*	3	Bull and Gillies (2007); Engler et al. (2020); Taib et al. (2021)
*Descriptive*	3	Goodnite (2013); Grandjean et al. (2021); Hagvall et al. (2016)
*Not Specified*	1	Henderson et al. (2017)
*Phenomenology*	1	Noyes (2000)
*Ethnography*	2	Oulton et al. (2018, 2020)
Quantitative	4	Borkovec et al. (1983); Borschuk et al. (2021); Madrigal et al. (2016); Phua et al. (2005)
Mixed methods	5	Nolan et al. (2014); Seliner et al. (2016); So et al. (2014); Woodson et al. (2015); Wright-Sexton et al. (2020)
Review	2	Mimmo et al. (2019); Shilling et al. (2012)

**Table 2.** Overview of included studies.

Author, date, country	Purpose	Sample with participant characteristics	Method/Study design	Concepts	Measurement tools/Outcome measures	Main results
Atout et al. (2017) Jordan	To explore the experience of decision-making in the care of children with palliative care needs in Jordan, from the perspective of their mothers	24 mothers, 12 physicians, 20 nurses	Qualitative (case study approach)	Decision-making for children in palliative care	Participant observation, semi-structured interviews	Mothers preferred to give the doctor the major role in deciding treatment. Mothers had little confidence in their ability to decide the best course and were fearful of making wrong decisions. Mothers also anticipated guilt related to decisions about their child. Sometimes mothers perceived a lack of good options impacting their decision
Borschuk et al. (2021), United States	To describe a novel behavioral health program and examine its impact on family parent engagement and psychological distress on a pediatric inpatient chronic ventilator unit	53 retrospective cases, 25 parents	Quantitative (post-intervention, cross-sectional study with historical control)	Family parent engagement, psychological distress	Qualitative parent engagement scale (QCES), NIH toolbox PROMIS measure, perceived stress questionnaire (PSQ)	No significant difference in family parent engagement pre/post intervention was noted. There was a significant increase in parent participation in medical rounds from 29.7% to 66.3%. Parents were rated as more engaged by staff and were more likely to complete education more quickly post-intervention. Parents distress decreased after psychotherapy
Brady et al. (2020) United States	To develop a comprehensive understanding of how families identify and communicate their child’s deteriorating health with the hospital-based health care team	28 parents (21 mothers, 2 fathers, 1 stepmother and 2 mother–father dyads)	Qualitative (thematic analysis)	Family identification and communication of their child’s deteriorating health	Semi-structured interviews, paper journaling	Six themes were identified: Parents’ expertise on their child’s baseline, informal pathways to navigate the complex and confusing system, importance of parental advocacy and persistence, parental roles, medical culture’s lack of support of partnership, and parents “running on empty” due to stress, fear, lack of sleep and loss of control in hospital
Bull and Gillies (2007) United Kingdom	To explore the views of hospitalized school-aged children with complex healthcare needs related to spiritual care	5 children	Qualitative (grounded theory)	Spiritual needs of school-aged, hospitalized children with complex healthcare needs	Semi-structured interviews	Main themes included: The role of the child’s relationships with family, friends and HCPs; the impact of the hospital environment; coping with invasive procedures; children’s views on their health and belief system
Engler et al. (2020) Germany	To explore how parents of children and adolescents with life-limiting conditions think about the hospital as place of care	13 parents (9 mothers, 4 fathers)	Qualitative (grounded theory)	Parents’ perceptions of the hospital as place of care	Unstructured narrative interviews	Parents reported feelings of vulnerability, heteronomy, and disablement associated with hospital care resulting from hospitals’ standardized care structures, over- and undertreatment, lack of continuity of care, hospital pathogens, lack of a palliative mindset, insensitive hospital staff, parental exclusion, the hospital stay as a permanent state of emergency, and a waste of limited life time
Goodnite (2013) United States	To analyze the concept of stress and present an operational definition of stress	Unspecified number of studies	Qualitative (concept analysis)	Stress	Literature review	Stress is a response to a stimulus or stressor that an individual perceives as being overwhelming and results in a non-biologic response that is not harmful to the individual
Grandjean et al. (2021) Switzerland	To explore the specific Pediatric Intensive Care Units (PICU)-related sources of stress, family functioning and needs of families of chronically critically ill patients during a PICU hospitalization	12 mothers, 8 fathers, 11 mother–father dyads	Qualitative (descriptive)	PICU-related sources of stress, perceived child’s quality of life	Semi-structured interviews	Five themes arose: High emotional intensity, PICU-related sources of stress, evolving family needs, multi-faceted family functioning, and implemented coping strategies
Hagvall et al. (2016) Sweden	To describe parental experiences of caring for their child with medical complexity during hospitalization for acute deterioration	9 parents (7 mothers, 2 fathers)	Qualitative (descriptive)	Parental needs, staff attitudes	Semi-structured interviews	The main theme was balancing the parent role with needing care. Parents felt they were in a vulnerable situation. They acted as the child’s ambassador to ensure their child is treated well and have staff respect their knowledge
Henderson et al. (2017) United States	To describe children’s experiences with PICU care for pediatric chronic critical illness	7 parents, 21 physicians, 15 nurses/nurse practitioners, 4 social workers, 1 other	Qualitative	Children’s’ experiences of care in the PICU	Semi-structured interviews	For the child, living in the PICU with acute care models was a poor match to meet needs. For families, there were acute-on-chronic stressors and barriers to visitation. It was also difficult to establish “PICU” goals of care and envision a transition to home
Madrigal et al. (2016) United States	To assess sources of support and guidance on which parents rely when making difficult decisions in the PICU and to evaluate associations with anxiety, depression, and positive and negative affect	86 parents (60 mothers, 26 fathers)	Quantitative	Sources of support and guidance	Sources of support and guidance instrument, the Positive and Negative Affect Scale (PANAS), the Hospital Anxiety and Depression Scale (HADS), the Adult Dispositional Hope Scale	Parents reported doctors, nurses, friends, extended family, and instinct as the strongest sources of support and guidance when making difficult medical decisions. Support groups, spiritual leaders, and church community ranked lowest
Mimmo et al. (2019) Australia	To systematically identify and synthesize peer-reviewed qualitative evidence of the parental experience of hospitalization with a child with intellectual disability	11 articles including children aged 0–18 years with intellectual disability, on in-patient units and parents	Systematic review	Parents’ experience of hospitalization with a child with intellectual disability	N/A	Five themes were identified: Being more than a parent, the importance of role negotiation, building trust and relationships, the cumulative effect of previous experiences of hospitalization and knowing the child as an individual
Nolan et al. (2014) United States	To describe and compare perceptions of parents (during their child’s hospitalization) with those of pediatric interns and pediatric hospitalists of long-term health-related quality of life (HRQoL) of children with severe disabilities	40 parents, 22 hospitalists, 20 pediatric interns	Mixed methods (observational, cross-sectional)	Perceptions of parents and physicians on long term health-related quality of life (HRQoL)	KIDSCREEN-10, semi-structured interviews	Parents rated their child’s HRQoL higher than physicians. Parents and physicians also expressed different goals for treatment. Parents expressed optimism despite uncertainty regarding their child’s future, whereas physicians anticipated increased medical complications and focused on parent burden
Noyes (2000) United Kingdom	To describe the views and experiences of young, ventilator-dependent people (and their parents) regarding the care they received, and to find out whether their needs were met during prolonged periods in intensive care units	18 children or youth (8 girls, 10 boys), 25 family members	Qualitative (phenomenology)	Meeting health needs, social needs environmental needs, and aspirations for the future	Focused interviews, some with draw/play techniques	Living in an intensive care unit is inappropriate and detrimental to the health and well-being of children. Four themes included: The experience of being dependent on a ventilator, the psychosocial impact of the intensive care unit, play and leisure, and rehabilitation
Oulton et al. (2020) United Kingdom	To identify what parents want from their relationship with healthcare professionals	9 parents of children and young people (CYP) with ID	Qualitative (ethnography)	Relationship between parents and healthcare professionals	In-depth interviews, participant observation	The overriding requirement was the need for a genuine partnership with professionals. Seven elements ideally characterize this partnership: Preparation, accessibility, reliability, trust, negotiation, expertise and respect
Oulton et al. (2018) United Kingdom	To understand the hospital-related needs and experiences of CYP with ID.	9 CYP with ID	Qualitative (ethnography)	Hospital-related needs and experiences of CYP with ID	Participant observation, tailored interviews, including art-based research methods	Five themes explained what is important to CYP with intellectual disabilities in the hospital: Little things make the biggest difference, eliminate unnecessary waiting, avoid boredom, routine and home comforts are key, and never assume
Phua et al. (2005) Australia	To evaluate differences in perceptions of inpatient care by parents of children with cerebral palsy (CP) compared to parents of able-bodied children	130 parents (40 parents of children with CP, 90 parents of children without disabilities)	Quantitative (observational, cross-sectional)	Perceptions of inpatient care Parental concern and dissatisfaction	Perceived Stress Scale (PSS-10) Instrument	Parents of able-bodied children were more satisfied with the hospitalization than parents of children with CP. Parents of disabled children had a much higher mean score on the Perceived Stress Scale
Ridner (2004) United States	To present a concept analysis of psychological distress, identifying antecedents, attributes, and consequences of psychological distress	Over 100 sources	Qualitative (concept analysis)	Psychological distress	Literature search	Distress is an emotional and non-specific biologic response to a perceived stressor or demand that has harmful effects on individuals
Seliner et al. (2016) Switzerland	To assess parental burden of care, satisfaction with family-centered care, and quality of life (HRQoL) of parents and the well-being of their hospitalized children with profound intellectual and multiple disabilities (PIMD) and determine the relationship among these factors	117 parents (98 mothers, and 19 fathers)	Mixed methods (observational, cross-sectional)	Parental burden of care, family-centered care, quality of life	Impact of Family Scale, Short Form Survey for HRQoL, Measure of Processes of Care, DISABKIDS Smiley questionnaire, semi-structured interviews	Parents indicated a substantial impact on burden of care and parental health-related quality of life. Significant correlations with the hospitalized children’s well-being were for burden of care and quality of life. Qualitative results showed parents struggling to safeguard their children and worrying most about the children’s well-being
Sexton et al. (2005) United Kingdom	To evaluate effectiveness of support required for children receiving home parental nutrition and their families by exploring the existing nursing homecare packages	20 families	Qualitative (thematic analysis)	Children receiving home parental nutrition, nursing home care packages	Semi-structured interviews	Parents reported isolation from their regular community due to high needs of hospitalized children
Shilling et al. (2012) United Kingdom	To synthesize qualitative research reporting the experience of disabled children as hospital inpatients and identify factors which affect their care	8 articles	Systematic review	Experiences of care of children in hospital	N/A	Communication between children and staff was a dominant theme. Other themes included emotions, particularly fears, the ward environment and confidence in staff
So et al. (2014) Canada	To examine parental perceptions of care of CMC during prolonged hospitalization in the context of an inpatient program [the Beanstalk Program]	20 parents	Mixed methods (post-intervention)	Parental experiences and perceptions during prolonged hospitalization	Measures of Processes of Care (MPOC-20) questionnaire, semi structured interview	Key themes included: Parents strive for positive and normal experiences for their child within the hospital; parents value the focus on child development in the midst of complex medical care; and appropriate developmentally focused education helps parents shift from feeling overwhelmed to instilling feelings of confidence and empowerment
Taib et al. (2021) Malaysia	To explore the challenges faced by parents with children who have complex neurological conditions, their coping strategies, needs, and expectations	11 parents (4 mothers, 7 fathers)	Qualitative (grounded theory)	Challenges faced by parents, coping strategies, needs and expectations	Semi-structured interviews	Eight domains of challenges included: Physical well-being, environment, relationship, financial, occupational, rational, mental, and spiritual
Woodson et al. (2015) United States	To assess the impact of specific child, parent, and family factors contributing to family hardiness	68 parents (43 mothers, 16 fathers and 9 guardians)	Mixed methods (observational, cross-sectional)	Family hardiness and resiliency	Family hardiness scale, semi-structured interviews	Four themes represented positive child coping: Staying active; physical comfort from a loved one; a positive attitude; participation in therapy at the hospital. Three themes represented negative child coping: Being noncompliant about using medical equipment; strong negative emotional reactions; lack of knowledge about his/her illness
Wright-Sexton et al. (2020) United States	To describe the experiences of parents and providers of children with chronic critical illness specifically around isolation during PICU admission	12 parents (9 mothers, 3 fathers), 7 PICU physicians, 8 nurse practitioners	Mixed methods (observational, cross-sectional)	Isolation during PICU admission	Semi-structured interviews, Center for the Epidemiological Studies of Depression Short Form, Medical Outcomes Study Social Support Survey	Parents did not feel medically isolated, although providers did. Parents self-reported adequate social supports but scored high on depression scales suggesting a disconnect between perceived and actual support

### Themes describing CMC and parents’ humanistic/illness related experiences

The themes developed during thematic analysis include: psychological impacts for parents, impacts on function of daily living, parents’ coping strategies for psychological well-being, impacts of hospitalization on CMC, CMC’ coping strategies, spirituality, and interventional studies. These themes are described in more detail below.

#### Psychological impacts for parents

Parents experienced psychological impacts when caring for their CMC during admission to an inpatient care unit (Seliner et al., 2016), including: stress, anxiety, distress, and worry.

##### Parents’ stress

Stress was the most frequently reported psychological impact for parents during admission to hospital with their CMC (Brady et al., 2020; Grandjean et al., 2021; Henderson et al., 2017). Stress is described as a response to a stimulus or stressor that an individual perceives as being overwhelming (Goodnite, 2013; Ridner, 2004). In one study, parents of children with Cerebral Palsy had significantly higher perceived levels of stress when compared to parents of neurotypical children admitted to hospital (Phua et al., 2005). Importantly, when stress became overwhelming, parents of CMC reported thoughts of suicide (Taib et al., 2021). See Table 3 for factors that cause parents stress.

**Table 3.** Factors that cause stress for parents.

Uncertainty	Uncertainty of health outcomes (Grandjean et al., 2021)
	Poor prognosis (Grandjean et al., 2021)
	Being confronted with end-of-life situations (Grandjean et al., 2021)
	Extended hospital stays (Henderson et al., 2017)
	Complications (Grandjean et al., 2021)
Child’s status	Physical appearance or behavior changes (Grandjean et al., 2021)
	Agitation and poor sedation management (Grandjean et al., 2021)
	Witnessing painful procedures (Grandjean et al., 2021)
	CMC connected to medical equipment (Grandjean et al., 2021)
Hospital environment	Overstimulating noises (Grandjean et al., 2021)
	Intimidating and gloomy atmosphere (Grandjean et al., 2021)
	Fear of contracting a contagious infection (Grandjean et al., 2021)
	Medical errors (Grandjean et al., 2021)
	Lack of normal home routines (Brady et al., 2020)

##### Parents’ anxiety, distress, and worry

Anxiety is described as a continuous experience where an individual experiences feelings of dread or impending doom (Bay and Algase, 1999), unlike stress, which is more of a short-lived response to a stressor. Parents of CMC reported that they felt panic and shock when unexpected events happened to their child (Oulton et al., 2020). They also felt anxiety when they were provided with limited information and not adequately prepared for what to expect during medical procedures (Oulton et al., 2020). Parents felt anxious about the safety of their children when they left them alone in the hospital with a nurse who was not familiar with them (Mimmo et al., 2019).

Distress is described as an emotional and non-specific biological response to a perceived stressor or demand that has harmful effects on individuals (Ridner, 2004). Parents of children in a pediatric inpatient chronic ventilator unit were found to have clinically elevated levels of distress using the Perceived Stress Questionnaire (PSQ) (Borschuk et al., 2021). However, after parents engaged in a behavioral health program their levels of distress decreased to normal ranges for the general population (Borschuk et al., 2021).

Worry is described as a process where individuals have a chain of negative thoughts or images that are uncontrollable and aimed at mental problem-solving an issue with an uncertain outcome (Borkovec et al., 1983). Parents of CMC reported that they often worried about their child (Taib et al., 2021). Parents worried about their decision to admit their children to the hospital and their children’s vulnerability, high dependency, limited ability to communicate, and fragility (Seliner et al., 2016). When children were more stable, parents felt more in control and experienced feelings of safety: when parents were not able to guarantee the well-being of their children, they felt exhausted and insecure (Seliner et al., 2016). Another cause for parents’ worry during hospitalization was the uncertainty of their children’s outcomes, which resulted in parents needing support and reassurance from the healthcare team (So et al., 2014). Parents of CMC in the PICU worried about their children growing and developing within an environment that they described as being a “constant horror” (Noyes, 2000).

##### Emotional rollercoaster

Parents described their experiences of being admitted to an inpatient care unit with their child as an “emotional roller coaster” (Seliner et al., 2016). Initially, upon diagnosis of their children, parents experienced shock and feeling as though “their world was unreal,” which was accompanied by feelings of fear due to uncertainty about their children’s future (Grandjean et al., 2021; Nolan et al., 2014). During admission, parents expressed that they felt vulnerable, out of control, and powerless (Engler et al., 2020). In one study, parents expressed that they were in a vulnerable position because they were dependent on HCP who often lacked the required knowledge and understanding of their children to provide adequate care (Hagvall et al., 2016). Parents also identified that they had feelings of insecurity because they were often left to advocate for and fend for themselves and their children (Hagvall et al., 2016). When parents felt disrespected by HCP they had feelings of anger (Grandjean et al., 2021).

Parents also reported feelings of isolation from their regular community because of the high needs of their hospitalized CMC (Sexton et al., 2005; Wright-Sexton et al., 2020). They also indicated that they felt lonely and bored in the hospital because it was just them and their child for long periods (Brady et al., 2020; Taib et al., 2021). As a result of being isolated and lonely, parents could experience emotions of fear and disorientation (Brady et al., 2020). During prolonged hospitalizations, parents of CMC longed for normalcy for both themselves and their children (So et al., 2014).

Guilt was another familiar emotion among parents of CMC (Taib et al., 2021); some parents would defer decision-making to physicians to avoid experiencing guilt if decisions had adverse outcomes (Atout et al., 2017). Some parents of CMC felt guilt and sadness when they believed they had “done wrong” as a parent or were suboptimal parents (Grandjean et al., 2021).

Parents reported that they experienced feelings of relief, joy, and gratefulness when a medical procedure went well or they were discharged (Grandjean et al., 2021). In one study, parents of children with severe disabilities rated their children’s quality of life higher than that of physicians (Nolan et al., 2014). They also noted that their CMC strengthened their family and expressed hopefulness regarding their children (Nolan et al., 2014).

#### Impacts on functions of daily living

Parents of CMC experienced strain in all areas of life when their child was admitted to the hospital. Parents described their hospital stay as a constant state of emergency, where they were unable to care for themselves (Brady et al., 2020; Engler et al., 2020). This included an inability to properly eat and sleep, which resulted in extreme levels of stress (Brady et al., 2020; Engler et al., 2020). Parents reported that they did not have a space for privacy and calm (Engler et al., 2020) and often woke in the night to provide care (e.g., suctioning) (Brady et al., 2020; Taib et al., 2021). Another source of exhaustion for parents during prolonged hospitalization was traveling long distances and other familial competing responsibilities, such as managing the home and caring for siblings (Engler et al., 2020; Henderson et al., 2017). Being in the hospital prevented families from living together and having normal daily routines, which they felt was a waste of the limited time they did have with their ill children (Engler et al., 2020; Seliner et al., 2016).

Parents of CMC expressed financial challenges, including nutritional costs, transportation costs to and from the hospital, and phone bills (Taib et al., 2021). Parents frequently took emergency leaves from work when their child was admitted to hospital, which resulted in unpaid leave and accumulated absences from work (Taib et al., 2021). Among parents of CMC in a Swiss University Hospital, parents experienced high levels of financial strain and fatigue, which was significantly correlated with parents’ health-related quality of life (Seliner et al., 2016).

#### Parents’ coping strategies for psychological well-being

It was important for parents to utilize strategies to cope with their psychological well-being and negative emotions. See supplemental file 3 for a table with strategies parents use to cope.

Parents received peer support from other parents of CMC (Taib et al., 2021). In the PICU environment, parents reported that they had an adequate support system. However, they lacked relationships with individuals who fully understood their experiences (Wright-Sexton et al., 2020). Only other parents of CMC could fully understand their life difficulties (Wright-Sexton et al., 2020) and provide them with support and comfort (Grandjean et al., 2021). They did note that some HCP understood the intricacies of their daily lives, but only other parents with CMC could truly sympathize with their struggles (Wright-Sexton et al., 2020).

Parents reported that doctors and nurses were a source of support when making difficult medical decisions (Madrigal et al., 2016). Parents could cope with their stressors and vulnerability when they had familiar staff who were sensitive to their needs and the care of their children because it increased their feelings of security (Hagvall et al., 2016). It was important to parents to negotiate roles with HCP to take breaks during their child’s hospital admission (Oulton et al., 2020). For example, they would go home to check on other children or make a cup of tea (Oulton et al., 2020). However, when parents were unable to negotiate this with HCP, they experienced increased stress of being unable to take a break (Oulton et al., 2020).

#### Impacts of hospitalization on CMC

Some CMC experienced negative responses during hospitalization, including fear, worry, sadness, depression, anxiety, boredom, and loneliness (Oulton et al., 2018; Shilling et al., 2012; Woodson et al., 2015). Factors that caused CMC’ negative responses are listed in Table 4.

**Table 4.** Factors influencing CMC’ negative psychological health.

Not engaging in normal everyday activities (e.g., school) (Oulton et al., 2018; Shilling et al., 2012; Woodson et al., 2015)	Painful procedures (e.g., blood draws, operations, taking medications) (Woodson et al., 2015)
Being separated from their family and disruption to normal routines (Shilling et al., 2012)	Lack of knowledge about their illness and diagnosis (Woodson et al., 2015)
Uncertainty about the reason for their admission (Oulton et al., 2018)	Unaware of upcoming medical procedures (Woodson et al., 2015)
Hearing other children crying (Oulton et al., 2018)	Not being included in care decisions (Oulton et al., 2018)
Poor sleep (Oulton et al., 2018)	Poor educational opportunities (Noyes, 2000)
Uncertainty of what was going to happen to them while in hospital (Oulton et al., 2018)	No access to appropriate systems of communications (Noyes, 2000)
Lack of play (Noyes, 2000)	

CMC’ extended hospitalizations in the ICU were described by parents as children “living in the ICU,” indicating that they require everyday childhood experiences during their prolonged stay (Henderson et al., 2017). Parents reported that their children wanted to interact and be included in interactions with others, in essence, to be treated like normal children (Nolan et al., 2014). This demonstrates CMC’ relationships were essential to their overall well-being (Nolan et al., 2014).

Parents highlighted that hospitalization had negative impacts on their children’s developmental growth, specifically due to decreased mobility, attachment to lines, illness symptoms, medical care, and prolonged stay (So et al., 2014). Parents also noted CMC’ quality of life corresponded to their ability to communicate and participate in care (Nolan et al., 2014).

CMC identified deficits in their basic needs when admitted to the hospital; CMC in the hospital noted that they need sleep and tasty food (Oulton et al., 2018). CMC reported that when admitted to the hospital they get thirsty, are cold at night, feel tired, and are disrupted by noises (Bull and Gillies, 2007; Oulton et al., 2018). One solution to this was a single bedroom and a comfortable bed (Oulton et al., 2018).

#### CMC coping strategies

CMC used a variety of methods to cope with their negative responses to hospitalization. CMC preferred to have the presence of their parent with them while in the hospital because their parents were a strong support (Bull and Gillies, 2007). See supplemental file 3 table with strategies CMC used to cope with stressors of hospitalization.

Parents aimed to create a positive and normal developmental environment for CMC in the hospital setting because they longed for normalcy despite their children’s illnesses (So et al., 2014). Parents reported in one study that avoiding disruption to routines and going at CMC’ pace helped CMC cope emotionally and physically with admissions to the hospital (Oulton et al., 2018). 

The manner in which HCP communicated and interacted with CMC during hospitalization was important for CMC’ ability to cope, including staff making children laugh and feel comfortable (Oulton et al., 2018). Relationships built between CMC and HCP were a source of support. However, it was noted that often times, HCP inflicted discomfort during medical procedures (Bull and Gillies, 2007; Oulton et al., 2018). CMC appreciated when nurses went out of their way to make children feel important (Oulton et al., 2018).

Factors that made it difficult for CMC to cope included: noncompliance with medical procedures, strong negative emotional reactions, and lacking knowledge about their illness and upcoming procedures (Woodson et al., 2015). When CMC were not able to cope they could become resistive and refuse or struggle to avoid medical procedures (Woodson et al., 2015). Specifically, children with intellectual disabilities were found to have a significantly reduced ability to cope with the emotional difficulties of prolonged hospitalizations (Oulton et al., 2018). This could result in the following: sadness, shouting, hitting, panic, and distress, which could further compromise the child’s physical health (Oulton et al., 2018). Other negative outcomes from CMC’ inability to cope included: becoming isolated, uncommunicative, or externalizing their emotions by being difficult with nursing staff (Woodson et al., 2015).

#### Spirituality

Spirituality is defined as a human characteristic expressed through beliefs, practices, and experiences to gain a connection with something that fosters meaning, promotes personal growth, contributes to the development of positive inner feelings, and assists in the formation of values (De Brito Sena et al., 2021). Parents reported the following spiritual challenges: uncertainty, self-value, finding meaning in life, not being judgmental, and feeling out of control (Taib et al., 2021). Some parents used spirituality to cope with their stressors, such as believing in God, accepting their situation/circumstances, living in the moment, being thankful, maintaining hope, engaging in prayer, and finding meaning in their hardships (Taib et al., 2021). Some CMC noted that God helped them during their difficult times and liked it when their parents would pray (Bull and Gillies, 2007). In one study, parents of CMC who were ranked as more spiritual had greater positive affect scores, and the parents who were ranked as less spiritual had increased depression (Madrigal et al., 2016). The authors of this study concluded that perhaps increased spirituality provided protection against depression and increased chances of sustaining positive affect (Madrigal et al., 2016).

#### Interventional studies

Two studies reported interventions to address experiences of care for CMC and their parents. Borschuk et al. (2021) implemented a novel behavioral health program among parents and CMC admitted to a chronic ventilator inpatient unit. It was offered in addition to standard care. It included structured multidisciplinary psychosocial rounds, evidence-based psychotherapy to address parents’ mental health, and support services and training by a psychologist for nurses, respiratory therapists, and nurse practitioners. The program was found to be significantly associated with decreased time for ventilator training, increased staff-rated parent engagement, and reduced parents’ distress.

Another interventional study was the Beanstalk Program, a program that centered on developmentally focused care for families in acute care settings (So et al., 2014). Parents in the program valued focus on developmentally appropriate care, and it helped them feel empowered rather than overwhelmed, instilling confidence during their transition home (So et al., 2014). Parents shifted from feeling overwhelmed by their child's care responsibilities to confidence in their role as a parent and advocate (So et al., 2014).

## Discussion

The aim of this article was to report findings from a large scoping review specific to CMC and parents’ experiences while receiving inpatient care that is related to their health and well-being. This review described how CMC and their parents struggled with their psychological and emotional well-being and how parents had trouble managing their functions of daily living. CMC and parents utilized a variety of coping strategies to manage their difficulties during admission. Specifically, some CMC and parents leaned into their spirituality, and two interventions were found to help parents cope.

Parents in this review experienced stress, anxiety, distress, and worry when their children were admitted to inpatient care units. This is echoed in MacKay et al. (2019) scoping review of parental experiences caring for hospitalized medically fragile infants; however, they also found parents had increased rates of depression. There are a variety of studies that demonstrate parents of hospitalized children have increased mental health conditions (Heuckendorff et al., 2021; Logan et al., 2020; Stremler et al., 2017).

In this review, parents experienced various emotions, ranging from fear, guilt, and uncertainty to joy and hope. Parents of infants who underwent cardiac surgery expressed that they alternated between emotions of sadness and despair to happiness and hope (De Man et al., 2020). Another study revealed that parents of children admitted to the PICU felt as though they were living in another world (Dahav and Sjöström-Strand, 2017). Parents suffer mentally and emotionally alongside their children when in the hospital; their psychological well-being should be considered in the care provided.

In this review, parents coped with their mental health in a variety of ways. This is similar to parents of infants admitted to the hospital for cardiac surgery who reported that they coped with their stress using cognitive strategies, engaging in hobbies (Kosta et al., 2015), and continuing to work (Clark and Miles, 1999). Parents of children with severe disabilities admitted to the hospital reported that they coped with their stress by leaning into supportive relationships, making meaning, normalizing, maintaining hope, and engaging in mental respite (Graungaard et al., 2011).

In this review, spirituality played a part in parents’ and CMC’ experiences when admitted to the hospital. According to Mueller (2010), children have an innate spiritual competence, and HCP should address the spiritual needs of children. In a cross-sectional study among children, parents, and pastoral care providers in the hospital setting, Freudnter et al. (2003) found that children required pastoral care for feelings of anxiety and fear, difficulty coping with pain, and family relationships. Specifically, parents required pastoral care for feelings of fear, guilt, and anxiety, difficulty coping with their children’s pain, questions about why their child was suffering, and questions about meaning and purpose (Freudnter et al., 2003). A barrier to pastoral care was HCP inability to identify patients’ spiritual needs, and all respondents felt the hospital could improve spiritual care services (Freudnter et al., 2003). Therefore, spiritual care of CMC and their families in the hospital setting is important; however, few studies were included in this review that spoke to this.

## Limitations

Screening of titles and abstracts was limited to articles published in English and did not include grey literature. Although measures were taken to ensure the comprehensiveness and exhaustiveness of the search, there is a chance that some relevant studies were not retrieved. This review included a variety of diagnoses that fall within CMC and parents’ demographics but were not considered in the synthesis. Therefore, given the wide range of included populations of both CMC and parents, these findings should be generalized with caution. Quality appraisals of included articles were not completed. Therefore, study biases were not considered in data analysis and synthesis. Grey literature was not searched and included. Thus, articles specific to this topic area could be missing. Few studies were conducted from CMC perspectives; thus, the children’s personal experiences may not be captured in this review.

## Implications for practice and future research

The comprehensive findings from this review should be disseminated to HCP who provide care for CMC. Specifically, a detailed understanding of causes of stress for parents, how they struggle with their emotional and mental health, and insight into their difficulty managing their functions of daily living. This could influence HCP’ approach to providing care. Also, awareness of coping strategies both CMC and parents used to manage their stress and mental health could help equip HCP with the skills required to support CMC and their parents. To do this, findings from this review could be incorporated into HCP training material and hospital educational sessions.

In the current review, all but one of the studies (Borschuk et al., 2021) that spoke to parents’ experiences with their psychological well-being were qualitative in nature. Given the plethora of validated measurement tools that can measure parents’ mental health, it is suggested that future studies be conducted that measure the quality of parents’ mental health when admitted to inpatient care units with their children. This will provide an understanding of the severity of their mental health struggles and, if done longitudinally, could demonstrate changes over time and identify triggers or events that are harmful to parents’ psychological health.

Only two studies included interventions targeted at supporting parents’ psychological health. However, both studies demonstrated promise in improving parents’ psychological outcomes. It is recommended that interventions targeted at supporting the psychological and emotional well-being of CMC and parents be developed, implemented, and evaluated in inpatient care units. CMC and parents noted leaning into their spirituality helped them cope with stress and uncertainty. However, few studies have been conducted in this area. Therefore, it is recommended that future research that explores spiritual care among CMC and their parents be conducted.

## Conclusion

Understanding patients and families’ experiences of care is valuable when attempting to improve care and services provided within healthcare systems. This review described in detail experiences of care for CMC and their parents specific to their health and well-being during hospital admission. Parents experience high levels of anxiety, distress, and worry, along with significant emotional disruption and challenges managing their functions of daily living. CMC experience negative emotions and mental health when admitted to hospital, often due to lack of social interactions and painful procedures. Both parents and CMC utilize a variety of coping mechanisms, including leaning into spirituality. Surprisingly, only two interventions have been conducted to support parents’ psychological health. This in-depth description of CMC and their parents’ experiences of care is beneficial to inform future research that further explores their experiences of care. This understanding, coupled with findings from this review, will aid in developing innovative care models and interventions to support CMC and their parents' health and well-being when admitted to inpatient care units.

## Supplemental Material

Supplemental Material - The health and well-being of children with medical complexity and their parents’ when admitted to inpatient care units: A scoping review Supplemental Material for The health and well-being of children with medical complexity and their parents’ when admitted to inpatient care units: A scoping review by Lyndsay Mackay, Tammie Dewan, Lauren Asaad, Francine Buchanan, K Alix Hayden, Lara Montgomery and Una Chang in Journal of Child Health Care

Supplemental Material - The health and well-being of children with medical complexity and their parents’ when admitted to inpatient care units: A scoping review Supplemental Material for The health and well-being of children with medical complexity and their parents’ when admitted to inpatient care units: A scoping review by Lyndsay Mackay, Tammie Dewan, Lauren Asaad, Francine Buchanan, K Alix Hayden, Lara Montgomery and Una Chang in Journal of Child Health Care

Supplemental Material - The health and well-being of children with medical complexity and their parents’ when admitted to inpatient care units: A scoping review Supplemental Material for The health and well-being of children with medical complexity and their parents’ when admitted to inpatient care units: A scoping review by Lyndsay Mackay, Tammie Dewan, Lauren Asaad, Francine Buchanan, K Alix Hayden, Lara Montgomery and Una Chang in Journal of Child Health Care
