# Supplementary material for: The health and well-being of children with medical complexity and their parents’ when admitted to inpatient care units: A scoping review
Source: J Child Health Care. 2025 Jan 29;30(1):188–210. doi: 10.1177/13674935241312299 (PMC12982579; doi:10.1177/13674935241312299)
Supplement: Supplemental Material - The health and well-being of children with medical complexity and their parents’ when admitted to inpatient care units: A scoping review [file sj-pdf-2-chc-10.1177_13674935241312299.pdf]

Database(s): **Ovid MEDLINE(R) and Epub Ahead of Print, In-Process, In-Data-Review & Other Non-Indexed Citations and Daily** 1946 to April 25, 2022
Search Strategy:

#	Searches	Results
1	Medically at-risk.tw,kf.	68
2	(complex adj4 (chronic condition* or health condition* or medical condition* or care need* or medical need* or healthcare need* or health care need* or health need* or disabilit*)).tw,kf.	3956
3	(medical* adj2 (complex* or fragile*)).tw,kf.	6089
4	(health adj2 (complex* or fragile*)).tw,kf.	5093
5	(Technolog* adj2 dependen*).tw,kf.	713
6	(ventilator adj2 (dependent* or assist*)).tw,kf.	1705
7	(mechanical* adj2 assist* adj2 ventilat*).tw,kf.	483
8	mechanically ventilated.tw,kf.	12772
9	medically fragile technology dependent*.tw,kf.	4
10	(special health care need* or special healthcare need*).tw,kf.	1857
11	polyhandicap*.tw,kf.	22
12	(profound adj4 (intellectual or disabilit*)).tw,kf.	1094
13	(life-limiting adj2 (condition* or disabilit*)).tw,kf.	649
14	chronic critical illness.tw,kf.	273
15	(intellectual disabilit* adj5 hospital*).tw,kf.	122
16	or/1-15	32653
17	Inpatients/	26925
18	Hospitalization/	127174
19	Child, Hospitalized/	7155
20	exp Hospitals/	302686
21	Adolescent, Hospitalized/	481
22	Hospitals, Pediatric/	14761
23	patient readmission/	21400
24	Intensive Care Units, Pediatric/	9058
25	((paediatric or pediatric) adj2 (icu or icu* or intensive care or ward* or unit*)).tw,kf.	19065

26	(picu or picus).tw,kf.	6252
27	inpatient*.tw,kf.	127173
28	(hospital* adj2 patient*).tw,kf.	116530
29	hospital*.tw,kf.	1494038
30	acute care.tw,kf.	25722
31	or/17-30	1701859
32	child/ or child, preschool/ or child/ or child, preschool/	2070859
33	exp pediatrics/	62361
34	(pediatric* or paediatric* or child* or preschool* or pre-school* or schoolchild* or schoolboy* or schoolgirl* or toddler* or boy or boys or girl* or pubescen* or juvenile* or teen* or youth* or high school* or adolesc* or pre-pubesc* or prepubesc*).tw,kf.	2170463
35	or/32-34	3000261
36	16 and 31 and 35	2714
37	limit 36 to yr="2000 -Current"	2443

Database(s): **Embase** 1974 to 2022 April 25
Search Strategy:

#	Searches	Results
1	Medically at-risk.tw,kf.	74
2	(complex adj4 (chronic condition* or health condition* or medical condition* or care need* or medical need* or healthcare need* or health care need* or health need* or disabilit*)).tw,kf.	5411
3	(medical* adj2 (complex* or fragile*)).tw,kf.	9887
4	(health adj2 (complex* or fragile*)).tw,kf.	6051
5	(Technolog* adj2 dependen*).tw,kf.	933
6	(ventilator adj2 (dependent* or assist*)).tw,kf.	2519
7	(mechanical* adj2 assist* adj2 ventilat*).tw,kf.	723
8	mechanically ventilated.tw,kf.	19708
9	medically fragile technology dependent*.tw,kf.	6
10	(special health care need* or special healthcare need*).tw,kf.	2198
11	polyhandicap*.tw,kf.	59
12	(profound adj4 (intellectual or disabilit*)).tw,kf.	1560
13	(life-limiting adj2 (condition* or disabilit*)).tw,kf.	978
14	chronic critical illness.tw,kf.	398
15	(intellectual disabilit* adj5 hospital*).tw,kf.	180
16	or/1-15	47521
17	hospital patient/ or hospitalized adolescent/ or hospitalized child/	209808
18	hospital/ or pediatric hospital/ or hospitalization/	845345
19	hospital readmission/	82655
20	pediatric intensive care unit/	10454
21	((paediatric or pediatric) adj2 (icu or icu* or intensive care or ward* or unit*)).tw,kf.	31826
22	(picu or picus).tw,kf.	13890
23	inpatient*.tw,kf.	214868
24	(hospital* adj2 patient*).tw,kf.	207978
25	hospital*.tw,kf.	2272249

26	acute care.tw,kf.	36443
27	or/17-26	2662339
28	child/ or juvenile/ or boy/ or girl/ or school child/ or adolescent/	2936052
29	preschool child/ or toddler/	593795
30	pediatrics/	85919
31	(pediatric* or paediatric* or child* or preschool* or pre-school* or schoolchild* or schoolboy* or schoolgirl* or toddler* or boy or boys or girl* or pubescen* or juvenile* or teen* or youth* or high school* or adolesc* or pre-pubesc* or prepubesc*).tw,kf.	2702862
32	or/28-31	3938137
33	16 and 27 and 32	4935
34	limit 33 to yr="2000 -Current"	4633
35	limit 34 to conference abstracts	1716
36	34 not 35	2917

Database(s): **APA PsycInfo** 1806 to April Week 3 2022
Search Strategy:

#	Searches	Results
1	chronically ill children/	415
2	Medically at-risk.tw,id.	53
3	(complex adj4 (chronic condition* or health condition* or medical condition* or care need* or medical need* or healthcare need* or health care need* or health need* or disabilit*)).tw,id.	1703
4	(medical* adj2 (complex* or fragile*)).tw,id.	1327
5	(health adj2 (complex* or fragile*)).tw,id.	1896
6	(Technolog* adj2 dependen*).tw,id.	280
7	(ventilator adj2 (dependent* or assist*)).tw,id.	80
8	(mechanical* adj2 assist* adj2 ventilat*).tw,id.	4
9	mechanically ventilated.tw,id.	242
10	medically fragile technology dependent*.tw,id.	2
11	(special health care need* or special healthcare need*).tw,id.	805
12	polyhandicap*.tw,id.	21
13	(profound adj4 (intellectual or disabilit*)).tw,id.	1285
14	(life-limiting adj2 (condition* or disabilit*)).tw,id.	235
15	chronic critical illness.tw,id.	19
16	(intellectual disabilit* adj5 hospital*).tw,id.	124
17	or/1-16	7617
18	hospitalized patients/ or patients/	40240
19	hospitalization/	8113
20	hospitals/	16348
21	hospital admission/	3116
22	intensive care/	4799
23	((paediatric or pediatric) adj2 (icu or icu* or intensive care or ward* or unit*)).tw,id.	1227
24	(picu or picus).tw,id.	478
25	inpatient*.tw,id.	53874
26	(hospital* adj2 patient*).tw,id.	17412

27	hospital*.tw,id.	171819
28	acute care.tw,id.	5265
29	or/18-28	233465
30	pediatrics/	28672
31	(pediatric* or paediatric* or child* or preschool* or pre-school* or schoolchild* or schoolboy* or schoolgirl* or toddler* or boy or boys or girl* or pubescen* or juvenile* or teen* or youth* or high school* or adolesc* or pre-pubesc* or prepubesc*).tw,id.	1074888
32	or/30-31	1075324
33	17 and 29 and 32	505
34	limit 33 to yr="2000 -Current"	450

CINAHL Plus with Full Text (Ebsco)

Search modes: Find all my search terms

#	Query	Results
S1	(MH "Child, Medically Fragile")	1,179
S2	TI “Medically at-risk” OR AB “Medically at-risk”	111
S3	TI ((complex N4 (“chronic condition*” or “health condition*” or “medical condition*” or “care need*” or “medical need*” or “healthcare need*” or “health care need*” or “health need*” or disabilit*))) OR AB ((complex N4 (“chronic condition*” or “health condition*” or “medical condition*” or “care need*” or “medical need*” or “healthcare need*” or “health care need*” or “health need*” or disabilit*)))	3,237
S4	TI ((medical* N2 (complex* or fragile*))) OR AB ((medical* N2 (complex* or fragile*)))	3,513
S5	TI ((health N2 (complex* or fragile*))) OR AB ((health N2 (complex* or fragile*)))	4,847
S6	TI (Technolog* N2 dependen*) OR AB (Technolog* N2 dependen*)	536
S7	TI ((ventilator N2 (dependent* or assist*))) OR AB ((ventilator N2 (dependent* or assist*)))	891
S8	TI (mechanical* N2 assist* N2 ventilat*) OR AB (mechanical* N2 assist* N2 ventilat*)	169
S9	TI “mechanically ventilated” OR AB “mechanically ventilated”	5,128
S10	TI “medically fragile technology dependent*” OR AB “medically fragile technology dependent*”	4
S11	TI ((“special health care need*” or “special healthcare need*”)) OR AB ((“special health care need*” or “special healthcare need*”))	1,599
S12	TI polyhandicap* OR AB polyhandicap*	5
S13	TI ((profound N4 (intellectual or disabilit*))) OR AB ((profound N4 (intellectual or disabilit*)))	839
S14	TI ((“life-limiting” N2 (condition* or disabilit*))) OR AB ((“life-limiting” N2 (condition* or disabilit*)))	549
S15	TI “chronic critical illness” OR AB “chronic critical illness”	150
S16	TI (“intellectual disabilit*” N5 hospital*) OR AB (“intellectual disabilit*” N5 hospital*)	121
S17	S1 OR S2 OR S3 OR S4 OR S5 OR S6 OR S7 OR S8 OR S9 OR S10 OR S11 OR S12 OR S13 OR S14 OR S15 OR S16	20,286
S18	(MH "Inpatients") OR (MH "Ventilator Patients")	87,006

S19	(MH "Infant, Hospitalized") OR (MH "Child, Hospitalized") OR (MH "Adolescent, Hospitalized")	6,226
S20	(MH "Hospitals") OR (MH "Hospitals, Pediatric")	76,202
S21	(MH "Readmission")	15,903
S22	(MH "Intensive Care Units, Pediatric")	6,737
S23	TI (((paediatric or pediatric) N2 (icu or icu* or "intensive care” or ward* or unit*))) OR AB (((paediatric or pediatric) N2 (icu or icu* or "intensive care” or ward* or unit*)))	9,860
S24	TI ((picu or picus)) OR AB ((picu or picus))	3,641
S25	TI inpatient* OR AB inpatient*	60,182
S26	TI (hospital* N2 patient*) OR AB (hospital* N2 patient*)	67,285
S27	TI hospital* OR AB hospital*	529,105
S28	TI "acute care" OR AB "acute care"	19,705
S29	S18 OR S19 OR S20 OR S21 OR S22 OR S23 OR S24 OR S25 OR S26 OR S27 OR S28	650,269
S30	(MH "Child") OR (MH "Child, Disabled") OR (MH "Child, Preschool") OR (MH "Minors (Legal)")	572,578
S31	(MH "Adolescence")	579,081
S32	(MH "Pediatrics")	21,437
S33	TI ((pediatric* or paediatric* or child* or preschool* or pre-school* or schoolchild* or schoolboy* or schoolgirl* or toddler* or boy or boys or girl* or pubescen* or juvenile* or teen* or youth* or “high school*” or adolesc* or pre-pubesc* or prepubesc*)) OR AB ((pediatric* or paediatric* or child* or preschool* or pre-school* or schoolchild* or schoolboy* or schoolgirl* or toddler* or boy or boys or girl* or pubescen* or juvenile* or teen* or youth* or “high school*” or adolesc* or pre-pubesc* or prepubesc*))	802,584
S34	S30 OR S31 OR S32 OR S33	1,208,859
S35	S17 AND S29 AND S34	2,017
S36	S17 AND S29 AND S34<br>Limiters - Published Date: 20000101-20221231	1,862

SRMedicalFragile Web of Science Search

Web of Science Core Collection
Editions = A&HCI , ESCI , CPCI-SSH , CPCI-S , SCI-EXPANDED , SSCI

#1

“Medically at-risk” or “mechanically ventilated” or “medically fragile technology dependent*” or “special health care need*” or “special healthcare need*” or polyhandicap* or “chronic critical illness” (Topic) or (complex Near/4 (“chronic condition*” or “health condition*” or “medical condition*” or” care need*” or “medical need*” or “healthcare need*” or “health care need*” or “health need*” or disabilit*)) (Topic) or (medical* Near/2 (complex* or fragile*)) (Topic) or (health Near/2 (complex* or fragile*)) (Topic) or (Technolog* Near/2 dependen*) (Topic) or (ventilator Near/2 (dependent* or assist*)) (Topic) or (mechanical* Near/2 assist* Near/2 ventilat*) (Topic) or (profound Near/4 (intellectual or disabilit*)) (Topic) or (life-limiting Near/2 (condition* or disabilit*)) (Topic) or (“intellectual disabilit*” Near/5 hospital*) (Topic)

#2

((paediatric or pediatric) Near/2 (icu or icu* or “intensive care” or ward* or unit*)) (Topic) or (picu or picus or inpatient*) (Topic) or (hospital* Near/2 patient*) (Topic) or hospital* or "acute care" (Topic)

#3

(pediatric* or paediatric* or child* or preschool* or pre-school* or schoolchild* or schoolboy* or schoolgirl* or toddler* or boy or boys or girl* or pubescen* or juvenile* or teen* or youth* or “high school*” or adolesc* or pre-pubesc* or prepubesc*) (Topic)

#2 AND #3 AND #1

#2 AND #3 AND #1 and 2021 or 2022 or 2020 or 2019 or 2018 or 2017 or 2016 or 2015 or 2014 or 2013 or 2011 or 2012 or 2000 or 2001 or 2002 or 2003 or 2004 or 2005 or 2006 or 2007 or 2008 or 2009 or 2010 (Publication Years)
