# Supplementary material for: The health and well-being of children with medical complexity and their parents’ when admitted to inpatient care units: A scoping review
Source: J Child Health Care. 2025 Jan 29;30(1):188–210. doi: 10.1177/13674935241312299 (PMC12982579; doi:10.1177/13674935241312299)
Supplement: Supplemental Material - The health and well-being of children with medical complexity and their parents’ when admitted to inpatient care units: A scoping review [file sj-pdf-3-chc-10.1177_13674935241312299.pdf]

Strategies Parents used to Cope

Maintaining good physical health (e.g., hydration, nutrition, good hygiene, sleep, and physical activity) (Grandjean et al., 2021; Taib et al., 2021)	Maintaining psychological health (e.g., psychotherapy, complementary therapies, positive thinking, and letting go) (Grandjean et al., 2021; Taib et al., 2021)
Maintaining social health (e.g., spending time with siblings and partners and activities with friends) (Grandjean et al., 2021)	Adapting and accepting their new reality (Grandjean et al., 2021) (Taib et al., 2021)
Partnering with their spouse (Taib et al., 2021)	Receiving help and support from family members (Taib et al., 2021)
Utilizing respite care (Taib et al., 2021)	Flexing work responsibilities and work hours (Taib et al., 2021)
Simplifying their life (e.g., cooking at home) (Taib et al., 2021)	Decreasing expectations (Taib et al., 2021)
supportive counselling helped them cope too (Hagvall et al., 2016)	

Strategies CMC used to Cope

Looking away from painful procedures (Bull and Gillies, 2007)	Thinking positively (Bull and Gillies, 2007; Woodson et al., 2015)
Playing games and with toys (Bull and Gillies, 2007; So et al., 2014; Oulton et al., 2018; Woodson et al., 2015)	Doing crafts (Bull and Gillies, 2007)
Going for walks (So et al., 2014)	Establishing routines (So et al., 2014)
Celebrating birthdays (So et al., 2014)	Watching television (Oulton et al., 2018)
Going to the playroom (Oulton et al., 2018)	Socializing with friends, patients, and staff (Bull and Gillies, 2007; Nolan et al., 2014)
Receiving comfort from family (Woodson et al., 2015)	Engaging in occupational and physician therapy (Woodson et al., 2015)
